# Choroid plexus aging: structural and vascular insights from the HCP-aging dataset

**DOI:** 10.1186/s12987-024-00603-y

**Published:** 2024-12-05

**Authors:** Zhe Sun, Chenyang Li, Jiangyang Zhang, Thomas Wisniewski, Yulin Ge

**Affiliations:** 1 Department of Radiology, NYU Grossman School of Medicine, 660 First Ave, Room 405, New York, NY 10016 USA; 2 Vilcek Institute of Graduate Medical Sciences, NYU Grossman School of Medicine, New York, NY USA; 3 Departments of Neurology, Pathology and Psychiatry, NYU Grossman School of Medicine, New York, NY USA; 4 Center for Cognitive Neurology, NYU Grossman School of Medicine, New York, NY USA

**Keywords:** Choroid plexus, Aging, Human connectome project, Perfusion function, Vascular degeneration

## Abstract

**Background:**

The choroid plexus (ChP), a highly vascularized structure within the ventricles, is essential for cerebrospinal fluid (CSF) production and metabolic waste clearance, crucial for neurofluid homeostasis and cognitive function. ChP enlargement is seen in normal aging and neurodegenerative diseases like Alzheimer’s disease (AD). Despite its key role of in the blood-CSF barrier (BCSFB), detailed studies on age-related changes in its perfusion and microstructure remain limited.

**Methods:**

We analyzed data from 641 healthy individuals aged between 36 and 90, using the Human Connectome Project Aging (HCP-A) dataset. Volumetric, perfusion, and diffusion metrics of the ChP were derived from structural MRI, arterial spin labeling (ASL), and diffusion-weighted imaging (DWI), respectively. Partial correlations were used to explore age-related ChP changes, and independent t-tests to examine sex differences across age decades. One-way ANOVA was employed to compare perfusion characteristics among ChP, gray matter (GM), and white matter (WM). Relationships between volume, perfusion, and diffusion were investigated, adjusting for age and sex. Additionally, the distribution of cyst-like structures within the ChP and their diffusion/perfusion MRI characteristics were analyzed across different age groups.

**Results:**

The ChP undergoes notable changes with age, including an increase in volume (*r*^*2*^ = 0.2, *P* < 0.001), a decrease in blood flow (*r*^*2*^ = 0.17, *P* < 0.001), and elevated mean diffusivity (MD) values (*r*^*2*^ = 0.16, *P* < 0.001). Perfusion characteristics showed significant differences between the ChP, GM, and WM (*P* < 0.001). Both the ChP and GM exhibited age-related declines in CBF, with a more pronounced decline in the ChP. A negative correlation was observed between the age-related increase in ChP volume and the decrease in CBF, suggesting compensatory dystrophic hyperplasia in response to perfusion decline. Cyst-like structures in ChP, characterized by lower MD and reduced CBF, were found to be more prevalent in older individuals.

**Conclusions:**

Our findings provide a detailed quantitative assessment of age-related changes in ChP perfusion and diffusion, which may affect CSF production and circulation, potentially leading to waste solute accumulation and cognitive impairment.

**Grant support:**

This work was supported in part by the NIH U01AG052564, P30AG066512, P01AG060882, RF1 NS110041, R01 NS108491, U24 NS135568.

## Background

The choroid plexus (ChP), a highly vascularized secretory structure in the brain’s ventricular system [1], plays key roles in immune function, maintaining the blood-cerebrospinal fluid (CSF) barrier (BCSFB), and clearing metabolic waste. The BCSFB, formed by specialized ChP epithelial cells connected by tight junctions [2], regulates the exchange of substances between the blood and the CSF, protecting the brain from harmful substances [3]. Blood plasma that flows through the fenestrated capillaries is filtered into the ChP interstitium or stromal tissue, primary due to hydrostatic pressure [4]. The ultrafiltrates including ions, nutrients, along with newly secreted CSF, are actively transported into the ventricles [2]. The ChP is the primary site for CSF production, crucial for cushioning the brain, regulating intracranial pressure, and driving CSF circulation. Histological studies have shown that the ChP undergoes age-related alterations, including shortened epithelial cells, impairment of tight junctions, stromal fibrosis, and calcium/iron deposition [5–7]. Changes in structure and blood perfusion of the ChP could potentially lead to impaired CSF production, accumulation of waste solutes, and increased permeability and impairment of the BCSFB [8, 9], which could further contribute to neurodegenerative processes in Alzheimer’s disease (AD) and other forms of dementia. Aging, a primary risk factor of dementia, leads to brain atrophy, loss of neurons and synapses, ventricular enlargement, and amyloid plaque accumulation. Given the role of ChP in AD, it is vital to investigate how normal aging affects the ChP in vivo and identify early diagnostic markers.

Prior MRI studies have predominantly focused on changes in ChP volume during normal aging and in neurodegenerative disorders such as AD, Parkinson’s disease, and multiple sclerosis [10–13]. Recent advancements in arterial spin labeling (ASL) techniques show promise for quantifying ChP perfusion and blood-CSF water exchange [14–17]. While pseudo-continuous ASL (pCASL) MRI studies indicate reduced ChP perfusion with age [10, 18], most are limited to animals or small sample sizes of in vivo human studies. The Human Connectome Project in Aging (HCP-A) provides a comprehensive dataset for studying normal aging, including advanced sequences with high-quality perfusion and diffusion MRI data for over 700 healthy adults aged between 36 and 100 years. This resource allows for a detailed analysis of cerebral blood flow (CBF), arterial transit time (ATT) [19], and microstructural changes in the ChP associated with aging. Previous studies, constrained by small sample sizes and resolution, may not fully capture the complexity of age-related changes in the ChP, especially in the absence of vascular evaluation.

In this study, we aim to conduct a comprehensive analysis of age-related changes in ChP measurements, with a specific emphasis on perfusion metrics, using a large adult lifespan dataset. We believe this will enhance our understanding of vascular degeneration and its relationship with the volumetric and microstructural changes associated with normal aging.

## Methods

### Participants from HCP-Aging dataset

The participants included in the HCP-A dataset are healthy volunteers, representing normal conditions for their age group. They have no identified pathological causes of cognitive decline, such as stroke, tumor, or clinical diagnosed dementia [20]. All participants provided informed, written consent for this Institutional Review Board (IRB) approved cross-sectional study. Data from participants older than 90 years were excluded due to the absence of precise age. More details regarding the cognitive assessments and additional inclusion and exclusion criteria for the HCP-A dataset have been described in the previous study [21]. In this study, 641 subjects (age: 36–90 years; mean ± SD = 60 ± 16 years; females/males = 359/282) who underwent the complete imaging protocol, including structural, perfusion, and diffusion MRI.

### Imaging acquisition

MR images of HCP-A were acquired with 3T using 32-channel head coil [20]. The imaging protocols included: (1) T1-weighted structural imaging was performed using multi-echo MPRAGE: TR/TI = 2500/1000 ms, TE = 1.8/3.6/5.4/7.2 ms, flip angle (FA) = 8°, voxel size = 0.8 mm isotropic, number of echoes = 4; (2) T2-weighted structural imaging was performed using a variable-flip-angle turbo-spin-echo (TSE) sequence: TR/TE = 3200/564 ms, turbo factor = 314, voxel size = 0.8 mm isotropic; (3) pCASL data were acquired using a single-shot 2D multi-band echo-planar imaging (MB-EPI) readout with the following parameters [22]: TR/TE = 3580/19 ms, partial Fourier = 6/8, labeling duration = 1500 ms, five post-labeling delays (PLDs) = 200 ms (control/label pairs = 6), 700 ms (control/label pairs = 6), 1200 ms (control/label pairs = 6), 1700 ms (control/label pairs = 10), and 2200 ms (control/label pairs = 15). A simultaneous multi-slice acquisition (SMS) with a multiband factor of 6 was used to achieve a 2.5 mm isotropic resolution (2.27 mm slice thickness plus 10% gap). The total acquisition time was approximately 5.5 min. Two 2.5 mm isotropic proton density (PD)-weighted equilibrium magnetization (M0) images were acquired at the end of scan for calibration. A pair of phase-encoding reversed spin-echo EPI scans that matched ASL data were acquired for distortion correction. More details of this advanced ASL acquisition were also described previously [23, 24]. (4) Diffusion imaging was performed using the multiband 2D spin-echo EPI with following parameters: TE/TR = 89.20/3230 ms, 1.5 mm isotropic, MB factor = 4, 28 non-diffusion-weighted (b0) images and 2 b shells (b = 1500/3000 s/mm^2^) with 92–93 directions per shell, acquisition time = 21 min. More detailed imaging parameters were described [20] and available on: https://www.humanconnectome.org/study/hcp-lifespan-aging/project-protocol/imaging-protocols-hcp-aging.

### Improvement of the choroid plexus segmentation

The initial processing of T1-weighted anatomical images was conducted automatically using the FreeSurfer-based HCP minimal processing pipelines (http://surfer.nmr.mgh.harvard.edu/) [25, 26]. However, due to the ChP having a small volumetric size and being immersed within the CSF, the segmentation obtained from FreeSurfer lacks sufficient accuracy. To overcome this limitation, a Bayesian Gaussian mixture model (GMM) was implemented in a stepwise manner (https://github.com/EhsanTadayon/choroid-plexus-segmentation). This method improved the differentiation of signal intensity between the CSF, the ventricular wall, and ChP voxels within the lateral ventricles, which in turn enhanced the accuracy of the segmentation [27].

### Image processing

The open-source HCP-ASL pipeline (https://github.com/physimals/hcp-asl) was utilized for the ASL-MRI image processing. The initial step involved correcting the label and control ASL images for a linear intensity variation, known as the banding effect, within each 10-slice band, caused by magnetization transfer (MT) effects. The data was also corrected for a slice-wise SMS-related saturation-recovery intensity variation by fitting the saturation recovery curve [28]. Motion correction was applied to improve saturation recovery fitting, and alignment of the de-banded ASL data [28]. The phase encoding-reversed spin-echo images were used for susceptibility distortion correction using TOPUP [29]. Following alignment with the pre-processed T1w image, perfusion analysis was conducted on ASL data, while the original 2.5 mm isotropic ASL voxel grid was preserved. After pre-processing, a general linear model (GLM) was used to perform the label-control subtraction. This helped rescale the perfusion-weighted signal and separate it from variations of through-slice motion [30]. The computation of CBF and ATT was carried out based on a Bayesian Inference for Arterial Spin Labeling (BASIL) toolkit implemented in FSL (version 6.0.5, Oxford, UK), with partial volume correction [31–33]. It allows fitting a two-compartment model to estimate the tissue and macrovascular contribution to the perfusion signal separately generating CBF and ATT maps [34, 35].

Diffusion Tensor Imaging (DTI) was preprocessed using MRtrix3 [36], including denoise, Gibbs’ ringing artifacts removal, distortion correction, and eddy current correction. After preprocessing, diffusion tensor was calculated and metrics including mean diffusivity (MD), and anisotropy (FA) were extracted afterwards.

**Fig. 1:**
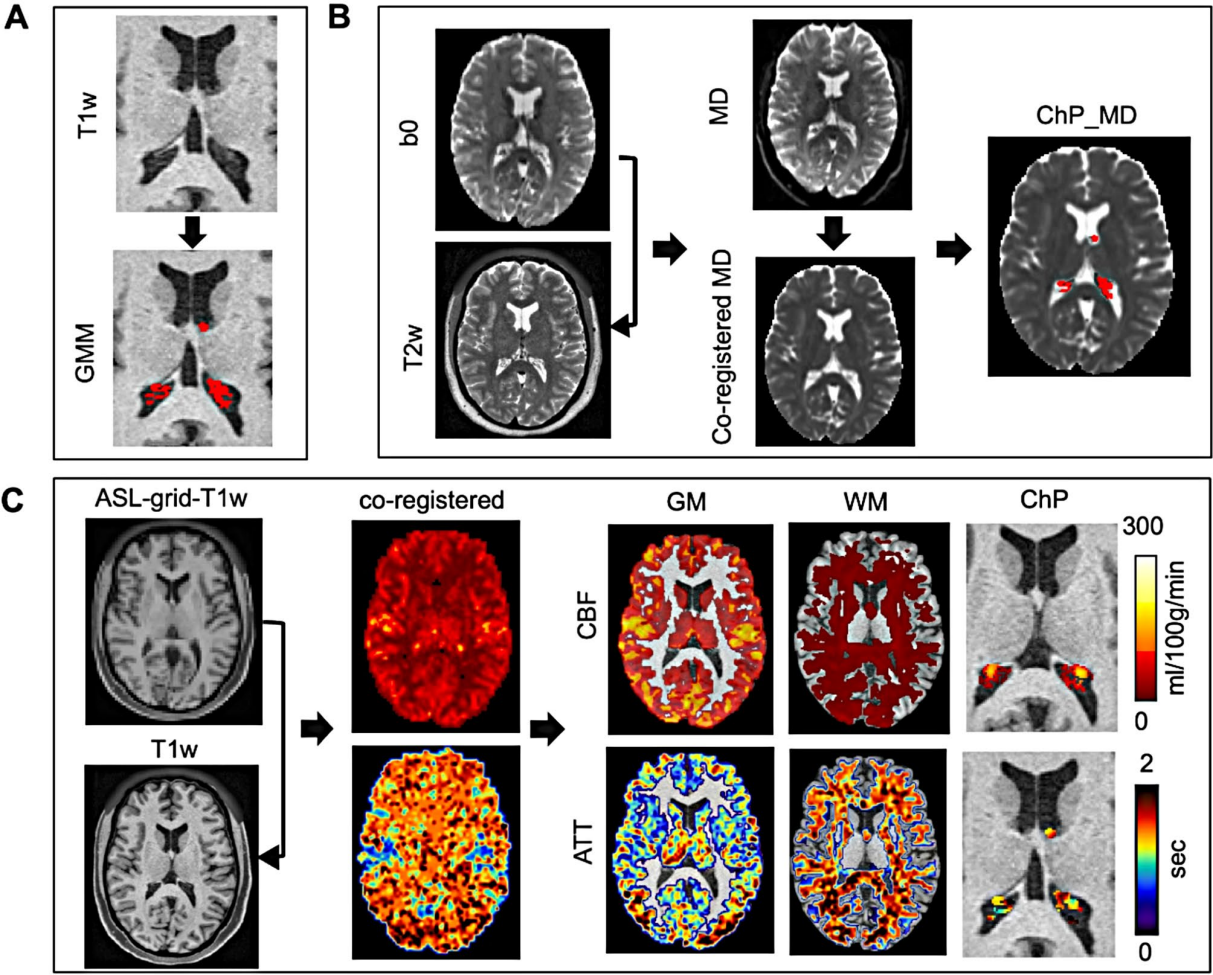
Image processing workflow for ChP analysis in HCP-A data. **(A)** Stepwise Gaussian Mixture Model (GMM) was performed for the ChP segmentation. **(B)** The b0 diffusion MRI image was registered to the T2w image, and the resulting transform matrix was applied to the MD map to calculate MD values within the ChP segmentation. **(C)** The ASL grid low resolution T1w data was registered to the original T1w image, and the transform matrix was applied to CBF and ATT maps to calculate CBF and ATT values within GM, WM, and ChP

The detailed image postprocessing steps are illustrated in Fig. 1. For each participant, the b0 image was registered to the T2w image, which exhibit similar image contrast, using 6-parameter linear rigid registration. The calculated transformation matrix was then applied to the corresponding MD map and the mean MD value within the ROI of ChP was calculated. CBF and ATT values was calculated in T1w space within the GM, WM, and ChP.

### Statistical analysis

We first employed the extra sum-of-squares *F* test to determine whether a linear or nonlinear model provided a better fit for the data. The test outcomes indicated a preference for a second order polynomial model for the volume and MD changes with aging, while a linear model was optimal for the correlation between age and CBF. The impact of age on the volume, CBF, and MD of the ChP was evaluated, accounting for sex as a covariate. The participants were roughly equally divided into three age categories: 36–49 years (33%), 50–69 years (37%), and 70–90 years (30%). Independent t-tests were conducted within each age bracket to discern the disparities between males and females in these measurements. One-way ANOVA analyses were employed to underscore the variations in perfusion attributes among the ChP, GM, and WM tissues. These analyses compared CBF and ATT values of different tissue types within each age group with Tukey’s multiple comparison correction. Partial correlations were computed between the CBF and ChP volume, and between the MD and ChP volume, after adjusting for age and sex. To reveal the difference between the ChP with and without the presence of cyst-like structures, independent t-tests were employed to compare the MD and CBF across different age groups. The Bonferroni correction was implemented to adjust for multiple comparisons, setting the significance level at an adjusted *P* < 0.016.

## Results

### Demographic characteristics

We exploited 641 healthy participants aged from 36 to 90 years old (60 ± 16 years; 282 males) from HCP-Aging dataset. Detailed demographic data and MRI measurements from different age quartiles can be find in Table 1.

**Table 1:** Detailed demographic data and MRI measurements

	All subjects 36–90 years (*n* = 641)	Q1 36–49 years (*n* = 211)	Q2 50–69 years (*n* = 243)	Q3 70–90 years (*n* = 187)
Age (years)	60 ± 15	43 ± 4	60 ± 6	79 ± 5
Sex (F/M)	359/282	123/88	136/107	100/87
Vol_ChP/_ICV (_*_10^− 3^)	1.93 ± 0.59	1.56 ± 0.45	1.86 ± 0.52	2.17 ± 0.65
MD (mm^2^/s*10^− 3^)	2.08 ± 0.73	1.86 ± 0.57	1.96 ± 0.64	2.50 ± 0.83
**CBF (ml/100 g/min)**				
ChP	58.85 ± 18.05	65.6 ± 17.6	58.2 ± 15.8	49.8 ± 15.3
GM	48.29 ± 11.42	51.9 ± 10.9	48.0 ± 9.7	43.1 ± 11.7
WM	21.64 ± 5.36	22.3 ± 5.1	21.7 ± 4.9	20.8 ± 6.1
**ATT (seconds)**				
ChP	1.39 ± 0.11	1.33 ± 0.1	1.40 ± 0.1	1.46 ± 0.1
GM	1.44 ± 0.12	1.36 ± 0.1	1.44 ± 0.1	1.51 ± 0.1
WM	1.48 ± 0.06	1.45 ± 0.05	1.48 ± 0.05	1.50 ± 0.06

### Age-related alterations in the choroid plexus

**Fig. 2:**
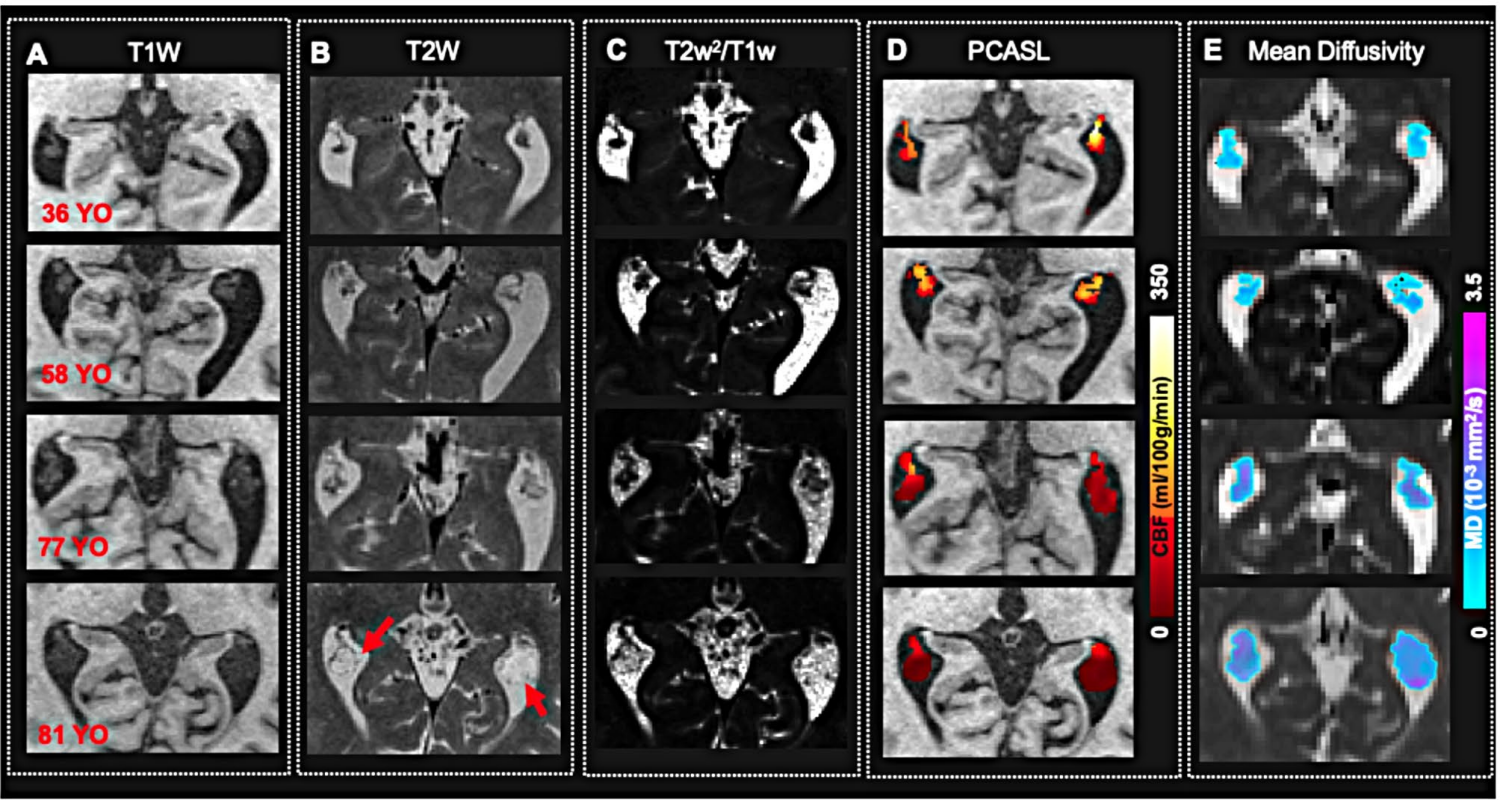
Representative images of ChP from individuals with different ages. **(A)** On T1w MRI, ChP volume increased with aging. **(B)** On T2 weighted MRI, the hypo-intensive flow-void vessels become sparser with cyst formation (red arrows) in older subjects. **(C)** By combining T1 and T2w data (T2w^2^/T1w), the contrast is enhanced, and the cyst delineation becomes easier. **(D)** CBF within the ChP on pCASL decreases with aging. **(E)** ChP MD values increase with aging

As shown in Fig. 2, T1w-MRI images show an enlargement of the ChP in elderly individuals. On T2w-MRI, the ChP appears less densely packed, with fewer hypo-intensive vessels and cyst formation. By combining T1w and T2w data and applying T2w^2^/T1w ratio, the ChP contrast is improved due to different T2 relaxation times between ChP blood and CSF. This results in a more defined outline of cyst-like structures. The outline of these cyst-like structures is improved on MD maps as well, which exhibit lower MD than CSF, making them more distinguishable.

**Fig. 3:**
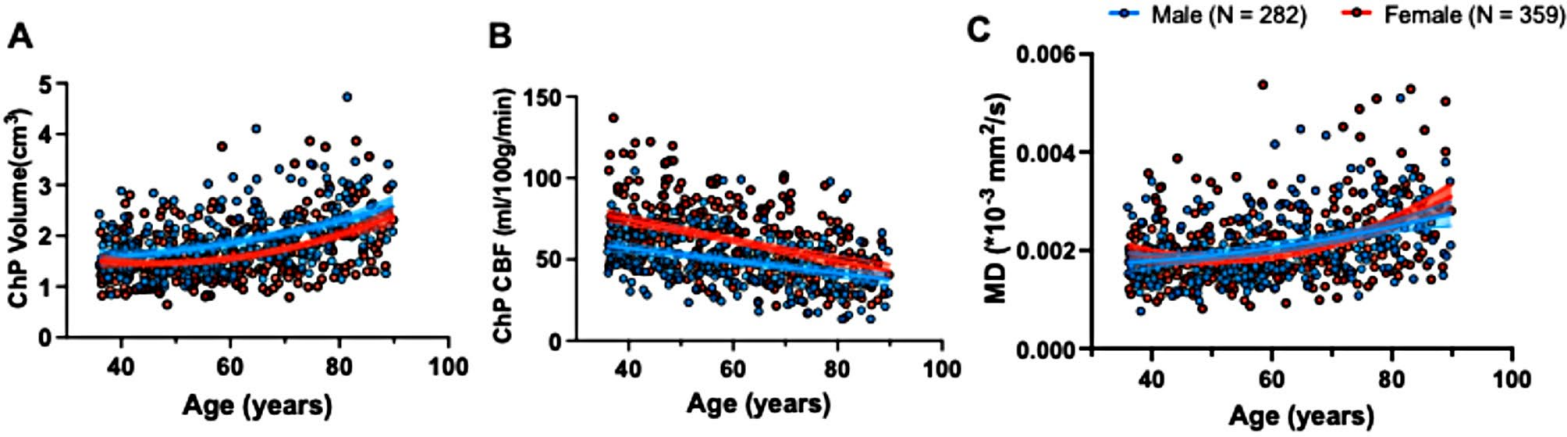
ChP changes associated with age. **(A)** The ChP volume showed a quadratic nonlinear relationship with age (*r*^*2*^ = 0.2, *P* < 0.001). Meanwhile, the ChP enlargement was faster in females than in males, particularly at later ages. **(B)** CBF within the ChP was negatively correlated with age (*r*^*2*^ = -0.17, *P* < 0.001), with female demonstrating a faster decline (β_female_ = -0.63, β_male_= -0.38). **(C)** The MD value showed a quadratic nonlinear relationship with age (*r*^*2*^ = 0.16, *P* < 0.001), the increase of MD was more rapid in females than that in males in the later lifespan

Figure 3 presents the volume, perfusion and diffusion measurements for all participants (*n* = 641) within the ChP, plotted as a function of age. After adjusting for the sex, the ChP volume showed quadratic relationship with age (*r*^*2*^ = 0.2, *P* < 0.001), with female demonstrating a faster enlargement ($female:{{dMD} \over {dAge}} = 7.42 \times {10^{ - 7}} \times Age - 7.2 \times {10^{ - 5}}$, $( male:{{dMD} \over {dAge}} = 2.08 \times {10^{ - 7}} \times Age - 7.6 \times {10^{ - 6}}$). The CBF within the ChP was negatively correlated with age (*r*^*2*^ = 0.17, *P* < 0.001), with female demonstrating a faster decline (β_female_ = -0.63, β_male_= -0.38). The MD value was positively correlated with age (*r*^*2*^ = 0.16, *P* < 0.001). The increase of MD was faster in females than that in male ($(female:{{dVol} \over {dAge}} = 4.7 \times {10^{- 4}} \times Age - 0.043$, $\,male:{{dVol} \over {dAge}} = 2.2 \times {10^{- 4}} \times Age - 0.009)$).

### Effects of sex on the measurements of the choroid plexus

**Fig. 4:**
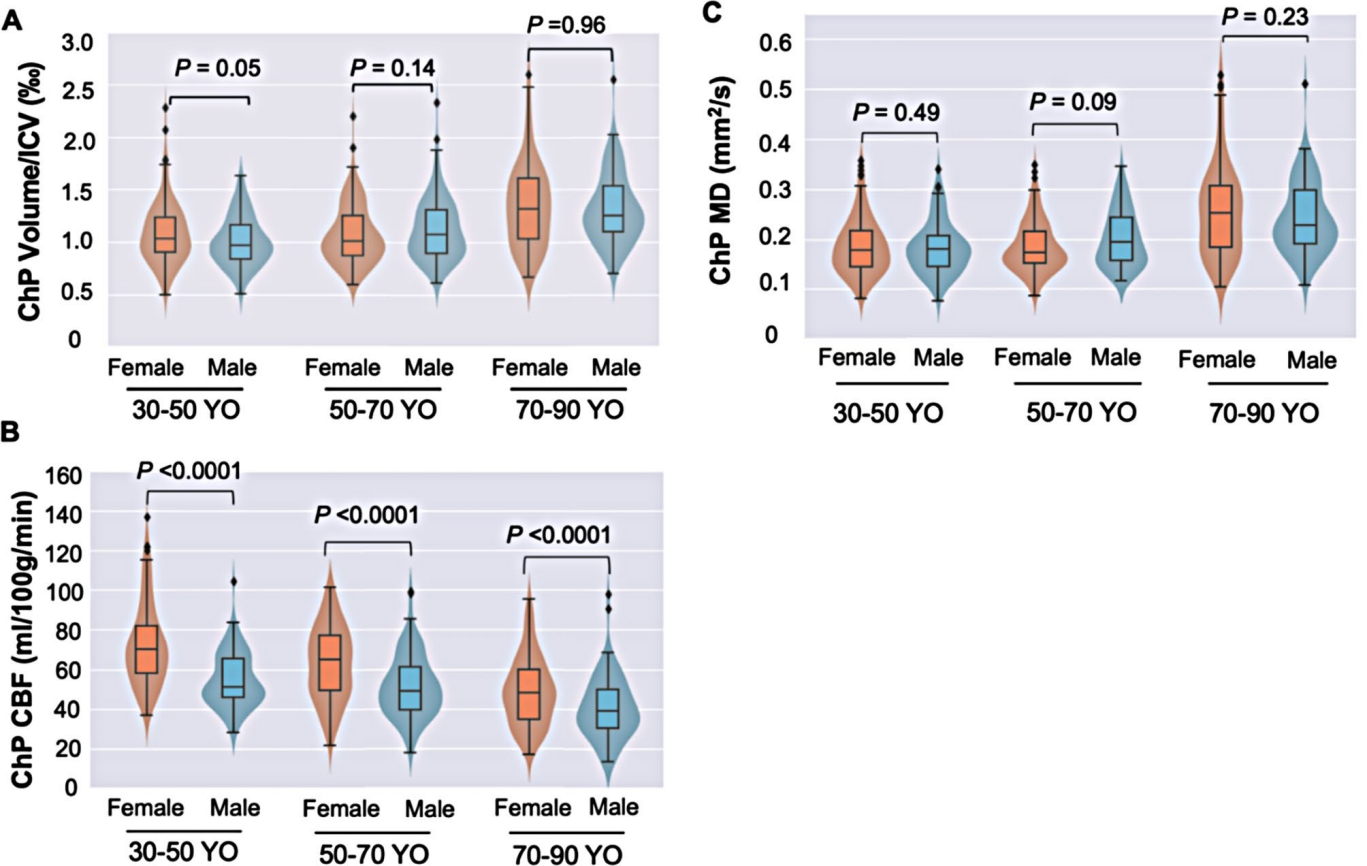
Effects of sex on the ChP alterations. **(A)** After adjusting the intracranial volume, there was no significant difference between males and females regarding the ChP volume. **(B)** The CBF within the ChP was significantly higher in the female subjects compared to the male subjects in all three age groups (*P* < 0.0001 for all). **(C)** There were no significant differences in ChP MD values for different age groups

As demonstrated in Fig. 3 on gender effects, females exhibited a faster rate of CBF decrease and MD increase compared to males in adult lifespan, in particular the later life. However, as shown in Fig. 4, after adjusting the intracranial volume, there was no significant difference between males and females in terms of the ChP volume for all age groups (*P*_30 − 50_ = 0.05, *P*_50 − 70_ = 0.14, *P*_70 − 90_ = 0.96). The CBF of the ChP was higher in the females compared to the males for all 3 age groups (*P*_30 − 50_ < 0.0001, *P*_50 − 70_ < 0.0001, *P*_70 − 90_ < 0.0001). No significant differences were observed between males and females regarding the MD values (*P*_30 − 50_ = 0.49, *P*_50 − 70_ = 0.09, *P*_70 − 90_ = 0.23).

### Perfusion characteristics of the ChP

One-way ANOVA followed by Tukey’s multiple comparison correction showed significant differences between the perfusion characteristics of ChP, GM, and WM. Detailed values are provided in Table 1. For all age groups, ChP has the highest CBF and the shortest ATT among the three tissue types, with the order of CBF: ChP > GM > WM, and ATT: ChP < GM < WM (*P* < 0.0001).

**Fig. 5:**
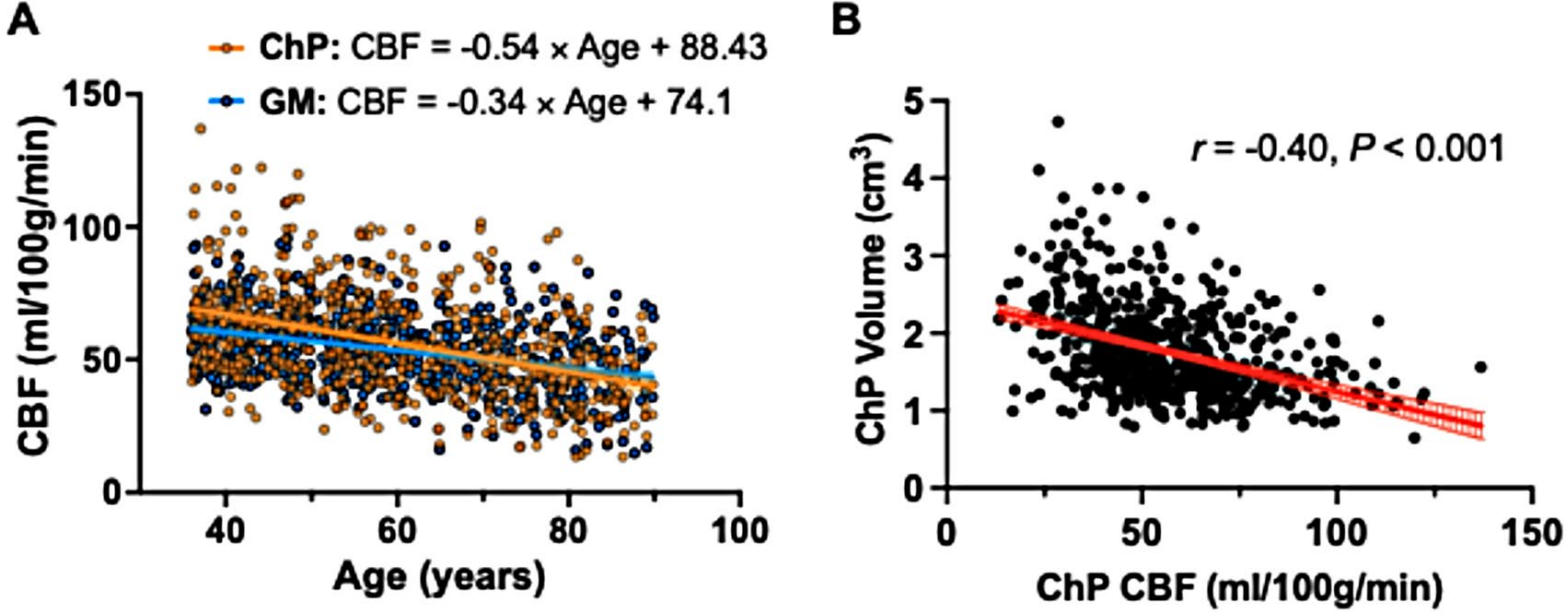
**(A)** ChP and cortical GM showed decreased CBF with advancing age (*r* = -0.40, *P* < 0.0001; *r* = -0.37, *P* < 0.0001, respectively). Age-related CBF decline of the ChP with age was faster compared to the GM (β_ChP_ = -0.54 vs. β_GM_ = -0.34). **(B)** After adjusting age and sex, negative correlation was observed between ChP volume and CBF (*r* = -0.40, *P* < 0.0001)

Both CBF in the ChP (CBF_ChP_) and GM (CBF_GM_) decreased with age (*r* = -0.40, *P* < 0.001; *r* = -0.37, *P* < 0.001, respectively). However, the rate of decline in CBF was faster in the ChP compared to the GM (β_ChP_ = -0.54, β_GM_= -0.34) (Fig. 5A). After adjusting for age and sex as covariates, a negative correlation was observed between ChP volume and CBF (*r* = -0.40, *P* < 0.001) (Fig. 5B). However, no significant correlation was found between CBF and MD (*P* = 0.1).

### Observation of cyst-like structures in the ChP

**Fig. 6:**
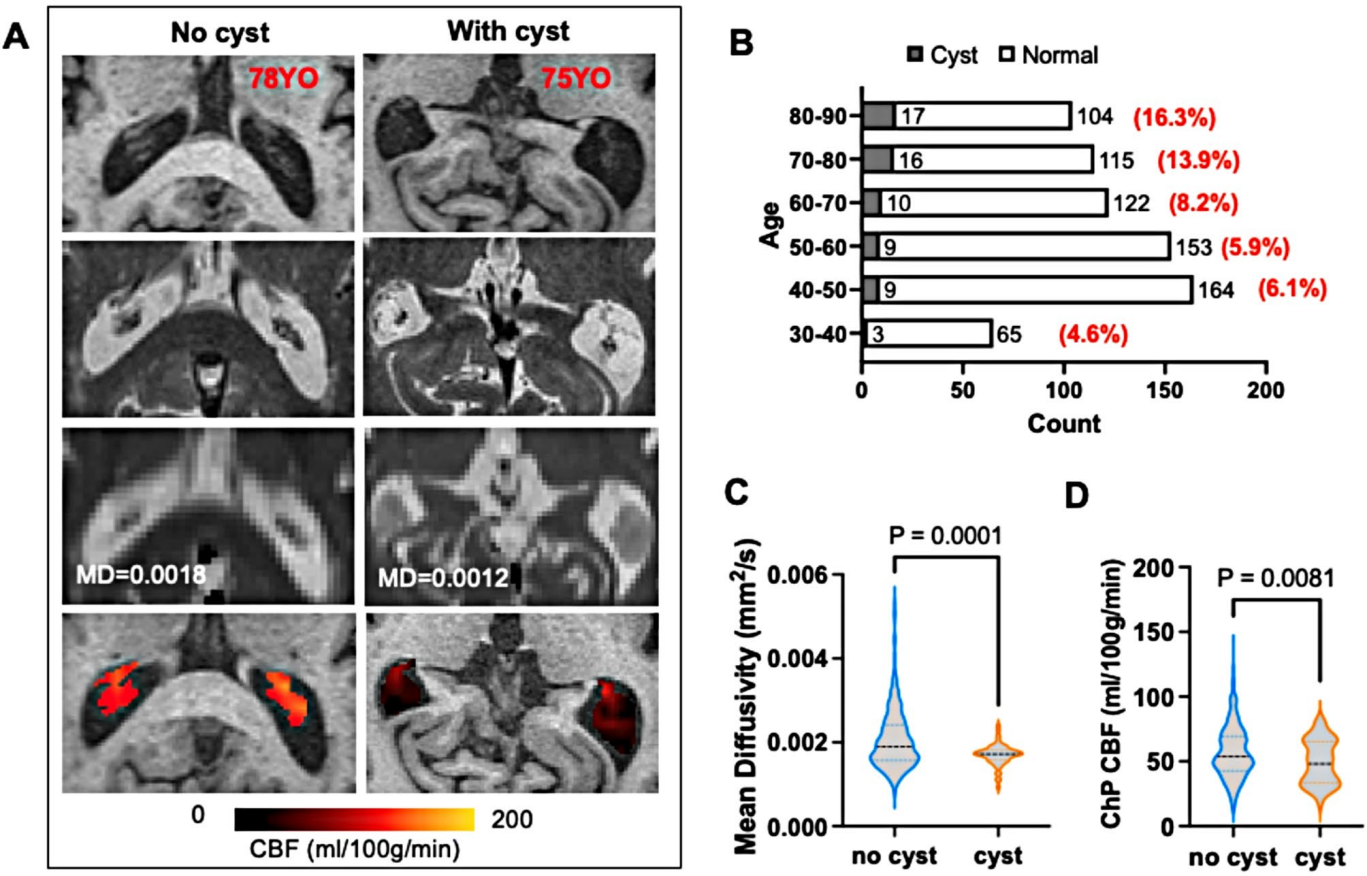
**(A)** Representative images from individuals with and without ChP cyst-like structures. MD values of the ChP with cysts were lower than those without cysts, demonstrating a restricted water motion. However, the CBF_ChP_ in subjects with cyst structures was lower than that without cyst. **(B)** The presence of cyst is getting more frequent with age. **(C)** The MD value in subjects with cyst structure ((1.69 ± 0.26) *10^− 3^) was lower than that without cyst ((2.06 ± 0.71) *10^− 3^) (*P* < 0.001). **(D)** The CBF is lower in the ChP with cyst (56.82 ± 20.03) than that without cyst (49.46 ± 17.15) (*P* = 0.0081)

Upon evaluating various imaging contrasts across all participants, we observed that cyst-like structures within the ChP are more common in the older population. These structures are distinguished by a sparse vasculature on the cyst wall and restricted diffusion or low MD values. As shown in Fig. 2, an 81-year-old subject with an enlarged ChP exhibits a cyst-like structure with hypo-to-iso-intense T1 and hyperintense T2 signals. These signals are similar with CSF intensity, but with lower MD than CSF. Figure 6A presents illustrative images from age-matched subjects with and without ChP cysts, demonstrating that cysts have lower MD and CBF values compared to the non-cyst ChP. The frequency of cyst occurrence, calculated for each decade, increases with age (Fig. 6B). There is a significantly decrease in CBF in ChP with cysts compared to non-cyst ChP in the 70–90 years age group (*P* = 0.005). Conversely, in the 30–50 years age group, CBF was slightly higher in ChP with cysts than in those without cysts (*P* = 0.01) (Fig. 6C).

## Discussion

In this research, we assessed age-related changes of ChP using perfusion and microstructural imaging in a large, well-characterized healthy cohort from the HCP-A datasets, aged between 36 and 90. In line with previous studies, we noticed an enlargement of the ChP associated with age, likely due to stromal fibrosis, dystrophic degeneration, calcification, and lipofuscin deposition [5]. Despite the increase in ChP volume with age, we noted a decrease in ChP blood flow. This seemingly contradictory relationship between blood flow and ChP size could indicate a hypertrophic response to chronic hypoperfused status. Unlike the ChP hyperplasia found in pediatrics, which leads to CSF overproduction [37], the enlargement of ChP in adults might be due to the accumulation of damaged cells and extracellular matrix components, or stromal fibrosis. Diffusion MRI revealed an increase in MD values with age, suggesting increased water mobility due to microstructural damage [38, 39]. The ChP exhibited the highest CBF, and shortest ATT compared to GM and WM, reflecting its highly vascularized nature essential for CSF filtration and secretion. The ChP showed a faster decline in CBF with aging compared to the GM. Despite the ChP’s abundant anastomoses, it might be more susceptible to age-related capillary loss. This could lead to inadequate CSF production rather than ischemic pathology, given its specific functions.

The glymphatic system, a recently identified waste removal system, facilitates a continuous exchange between CSF and interstitial fluid (ISF) via convective influx of CSF along the periarterial space [8]. Although the role of ChP in the glymphatic system remains largely unclear, the production of CSF and its subsequent entry along the perivascular space is considered crucial for ISF-CSF exchange and clearance function. The glymphatic influx serves as an essential route for the distribution of electrolytes, macromolecules, and other larger compounds that predominantly enter the brain through the BCSFB at the ChP, as hypothesized [40]. The ChP is comprised of a monolayer of polarized secretory epithelial cells whose primary role is the production and secretion of 60–75% of total CSF [41]. The fenestrated capillaries permit the rapid diffusion of fluid and small molecules into the stroma and allows for the rapid production of CSF, in which, the total volume (150 ml) of CSF is circulated and replaced approximately 3 to 4 times per day for a normal adult [42]. While the CSF volume seems increased due to the large ventricle size primarily caused by neurodegenerative tissue loss, the CSF turnover rate decreased (slow circulation) in the elderly [43]. CSF secretion is a complex process with mechanisms that remain partially understood. The prevailing model describes CSF production as occurring in two distinct stages: passive ultrafiltration of blood plasma through the fenestrated capillaries of the basal lamina, followed by the active transport of fluid and solutes across the epithelial barrier, both driven by an osmotic gradient from blood flow [44–46]. Further, recent evidence suggests that a significant portion of CSF secretion occurs via water transport independent of the traditional views of transchoroidal blood-CSF osmotic gradient [47]. This process is believed to involve transcellular transporters, including aquaporin-1 (AQP1), which is predominantly expressed in the apical membrane of ChP epithelial cells, as well as Na+/K+-ATPase, bicarbonate transporters, and glucose transporter-1 (GLUT1), primarily located in the capillary basement membrane [48–51]. In the aged and AD populations, vascular degeneration and the shortening ChP epithelial cells due to aging are thought to reduce the expression or the functions of AQP1 and GLUT1, thereby diminishing the capacity for CSF production [52, 53].

Recently, Evans et al. [15] and Lee et al. [54] applied a novel ASL-based sequence with a long TE in animal studies, specifically measuring the delivery of labeled arterial blood water from the ChP into ventricular CSF following vasopressin administration. They observed decreases in both ChP perfusion and CSF secretion, indicating a tight coupling between ChP perfusion and CSF secretory function, consistent with findings from earlier invasive physiological study [55]. We consider the ChP as the primary site of CSF secretion and a key driver of CSF-ISF circulation within the brain [40]. A recent human study using both ASL and phase contrast (PC)-MRI demonstrated that increased ChP perfusion is associated with greater net CSF flow through the aqueduct increased in the caudal (positive) direction, indicating a relationship between ChP perfusion in the lateral ventricles and downstream CSF flow at the level of the aqueduct of Sylvius in healthy adults [18]. Human and animal studies indicate that net CSF flow or blood-water delivery to the ventricles declines with age [15, 56]. Furthermore, aging mice exhibit elevated levels of Aβ1–40 and Aβ1–42 in the cortex and hippocampus [57], likely resulting from reduced CSF turnover and impaired CSF-ISF exchange, underscoring the critical association between CSF dynamics and waste clearance. Although these studies suggest that reduced ChP perfusion or blood flow may contribute to a reduced rate of CSF secretion, further direct evidence is needed to better elucidate their relationship.

The observed decrease in ChP perfusion with age is consistent with findings from Alisch et al. [10], who reported a decrease in ChP perfusion with aging using single post-labeling delay pCASL MRI. Vascular aging can lead to increased vessel tortuosity due to changes in the mechanical properties of the vessel walls and the pulsatile hemodynamic forces acting on them. This is particularly evident in structures with rich vascular network, such as ChP. Over time, these vessels undergo structural and functional alterations, including the weakening of the vessel wall and a decline in elasticity. This process, often referred to as arterial stiffening, results from the accumulation of collagen, fragmentation of elastin fibers, and the presence of calcification [58]. As a result of these changes, the vessel wall’s capacity to withstand mechanical stress decreases, making the vessel more prone to deformation due to the pulsatile blood flow, and higher pressure on the tortuous vessel wall during each cardiac cycle over decades. To our knowledge, there has been no ASL-MRI study specifically investigating ChP pulsation. However, 4D flow MRI has been used to examine CSF pulsation patterns within the ventricles, which indirectly reflect arterial pulsatility but are not specific to the ChP. CBF dynamics, including low-frequency vasomotion driven by neuronal activity [59, 60], respiratory-induced CBF-CSF motion coupling [61], and cardiac-related vessel wall pulsation [62], may serve as pumping mechanisms that propel CSF through the subarachnoid space (SAS) and along PVS to facilitate CSF-ISF exchange within the parenchyma or directly within the PVS [63]. CSF bulk flow is pulsatile and can be affected by conditions like arterial hypertension or arterial wall stiffening, which may disrupt the perivascular pump and slow CSF transport [64]. Immunostaining studies have shown tortuosity changes in arterioles and capillaries within the aged ChP [65]. Age-related vascular tortuosity has also been observed in both large [66] and small [67] brain arteries in humans. Together with findings from our prior in vivo 7T-MRI human study [68] showing sparser ChP vasculature, these vascular changes may impact CSF secretion and dynamics, potentially influencing neurological function.

The decline in CBF, in conjunction with ChP enlargement, may reflect a compensatory mechanism involving dystrophic hyperplasia triggered by reduced perfusion [69]. If glymphatic CSF to interstitial flow is compromised, the ChP could potentially increase CSF production to compensate and facilitate the glymphatic clearance of waste products from the interstitial space [69]. While the exact mechanism remains to be established, we propose that the ChP might instead undergo non-functional stromal expansion involving stimulation from immunoreactivity [7, 70], though the exact mechanism remains unclear.

The observed increase in MD with aging could potentially signify a loosening of the ChP structure and a decrease in the microstructural integrity of the BCSFB. MD measures the overall diffusion and motion of water molecules, with higher MD values indicating greater water mobility. This finding is consistent with a previous study [10], that reported decreased FA, and increased T1 and T2 values. However, in our study, we did not find a significant correlation between FA values and age, nor any sex-based differences in MD values. This discrepancy could be due to variations in segmentation and registration methodologies. Furthermore, the ChP is not a highly anisotropic structure, and FA is less sensitive to its microstructural changes compared to MD.

It is worth noting that the CBF in the ChP is significantly higher in females than in males across all age groups. This pattern aligns with previous studies that indicate greater whole-brain and GM perfusion in females compared to males [71, 72]. This phenomenon could potentially be attributed to the effects of estrogens [73]. Furthermore, the rate of CBF reduction in the ChP with age was faster in females. While no significant sex differences were observed in the ICV-adjusted ChP volume and MD values, the rates of age-related changes for ChP enlargement and MD values were higher in females. This suggests a greater susceptibility in females to age-related perfusion and microstructural changes in the ChP. It has been suggested that males and females with AD exhibit different cognitive and psychiatric symptoms, and females demonstrate faster cognitive decline after diagnosis of AD dementia [74]. In addition, a significantly weaker glymphatic function, as indicated by neuronal activity-CSF coupling strength, has been observed in females than in males [75]. Taken together, these sex effects within the ChP may contribute to the sex-specific clinical and pathological findings of age-related neurodegenerative diseases, considering the interactions between ChP, the glymphatic system, and AD.

Despite the age-associated increase in MD, a higher prevalence of cyst-like structures, characterized by restricted water movement and thus lower MD, was noted in older individuals. Based on our observation, cyst formation occurs specifically within the ChP in this large-scale HCP-Aging dataset. This phenomenon is likely attributed to the unique characteristics of the ChP, including its high vascularization and relatively loose stromal tissue structure [68]. Surgical findings have confirmed that ChP cysts contain a serous fluid akin to CSF, with the protein concentration in the cyst fluid being slightly elevated compared to that of CSF [76]. Histologically, the thickened walls of the cysts were composed of abundant connective tissue with blood vessels and foci of calcification [77]. The contents of these connective tissue-lined cysts was gelatinous and tested positive for fibrinogen and albumin immunohistochemical staining, indicating a highly proteinaceous composition [77]. An MRI study employing oscillating gradient spin-echo (OGSE) DWI with shortened diffusion time suggested that the lower ADC values of the ChP cysts, compared to free CSF [78], imply the existence of spatially restricted diffusion and increased viscosity of the cysts. Due to their various lipid and blood components, these structures can present heterogeneous contrast on MR images. Without histological validation, differentiation age-related cystic changes with ChP xanthogranulomas (XGs) with psammoma bodies can be challenging, as both exhibit similar characteristics on DWI. XGs are benign lesions with an autopsy incidence of 1.6–7%, which is lower than the rate of cysts observed in our study. It has been validated that the cyst wall consists of rich connective tissue and foci of calcification. The restricted water movement in aged ChP could be due to calcium deposits, cholesterol crystal accumulation, or gelatinous fluid. Although the formation of these cysts is generally benign, their continued growth could result in the gradual destruction of choroidal villi and increased dystrophic hyperplasia [79]. Regardless of the cyst type, we propose that these structures could negatively impact the vascular component of the ChP and potentially disrupt its homeostatic function in the brain.

From a technical standpoint, the HCP-A dataset provides high quality pCASL MRI with multiple PLDs, allowing for simultaneous measurements of ATT and CBF. While segmented 3D PCASL is recommended for ASL in clinical applications, it is susceptible to head movement, especially in older individuals. The ASL sequence in the HCP-A protocol incorporates cutting-edge developments in data acquisition like using multi-band echo planar imaging (MB-EPI) multi-slice (SMS) imaging to reduce acquisition time and minimize motion artifacts in whole brain perfusion imaging. However, the application of SMS may result in signal voids at imaging band edges. According to Li et al. [23], the geometry factor (g-factor) and the total leakage factor (TLF)-induced confounding effects, have a minimal impact on the CBF estimation.

Certain limitations of the study should be acknowledged. Firstly, the ChP is immersed within the CSF, which necessitates consideration of partial volume effects (PVEs). However, we consider that the large dataset and high-quality images used in this study could help counterbalance these PVEs. Furthermore, the application of the GMM method to improve segmentation could also aid in bias reduction. A combination of T1w and T2w images (T2w^2^/T1w) was also used to augment the tissue contrast between CSF and ChP, sharpening their boundary. The enhanced vascular contrast on this ratio image is primarily due to the hypointense signal of the vascular glomus on T2, caused by flow voids, and slightly hyperintense T1 signal relative to CSF, which enhances the tissue contrast. The T2 relaxation time of the fluid within cyst-like structure might differ from that of the ChP blood and CSF. Therefore, the use of the T2w^2^/T1w ratio could amplify the contrast between these compartments. This improves visualization of the cyst-like structures and enables more accurate neuromorphometric analyses [80]. Our prior study using high-resolution contrast-enhanced MRI on 7T revealed detailed ChP anatomy, showing age-related decrease in vascular density [68] and corroborating the current study’s findings of reduced CBF with age. Secondly, this study does not include clinical data such as medication usage and vascular risk factors (i.e., blood pressure, glucose level, and cholesterol level). These factors could potentially influence vascular health and cognitive function [21]. However, it is crucial to highlight that, based on prior research, aging is the most significant risk factor for numerous neurodegenerative disorders. For example, we did not observe significant effects of these factors on the ChP in our prior study [68]. Future research should include these parameters for a more comprehensive evaluation. Thirdly, the HCP Aging dataset did not provide direct measurements of CSF flow, such as through PC-MRI. However, our findings, combined with previous research, suggest an interrelationship between ChP perfusion function and CSF production. Future studies with larger sample sizes and a wider age range, including direct CSF flow measurements, are needed to establish their correlation.

## Conclusion

The large-scale HCP-Aging dataset, covering a wide age range of adult lifespan, provides high-quality structural, diffusion, and perfusion MR images. Our findings show that the ChP undergoes age-related changes, including enlargement, CBF decline, and elevated MD. These alterations may result from stromal expansion, reduced vascular density, and compromised BCSFB integrity, potentially leading to insufficiency in CSF production, waste accumulation, and impaired exchange between CSF and ISF in the glymphatic system, ultimately affecting cognitive function.

## Data Availability

No datasets were generated or analysed during the current study.
